# Supplementary material for: Vitamin B12 alleviates spliceosomopathy via phospholipid remodeling
Source: Nat Commun. 2026 Aug 7;17:8011. doi: 10.1038/s41467-026-76295-9 (PMC13451374; doi:10.1038/s41467-026-76295-9)
Supplement: Supplementary file 1 [file 41467_2026_76295_MOESM1_ESM.pdf]

Supplementary Information for

Vitamin B_{12} alleviates spliceosomopathy via phospholipid remodeling

Jonathan Kölschbach[1], Hormos Salimi Dafsari[1,2,3,4], Emily Baum[1,2,3,4], Anna Löhrke[1], Chun Kew[5,6], Ivan Dikic[5,6], Wenming Huang[1,7,*] & Adam Antebi[1,2,*]

[1]Max Planck Institute for Biology of Ageing, 50931 Cologne, Germany

[2]Cologne Excellence Cluster on Aging and Aging-Associated Diseases (CECAD), University of Cologne, 50931 Cologne, Germany

[3]Department of Pediatrics, Faculty of Medicine and University Hospital Cologne, University of Cologne, 50937 Cologne, Germany

[4]Center for Rare Diseases, Faculty of Medicine and University Hospital Cologne, University of Cologne, 50937 Cologne, Germany

[5]Buchmann Institute for Molecular Life Sciences, Goethe University, 60438 Frankfurt, Germany

[6]Institute of Biochemistry II, Faculty of Medicine, Goethe University, 60590 Frankfurt, Germany

[7]Institute of Clinical Genetics and Genomic Medicine, University Hospital of Würzburg, 97080 Würzburg, Germany

*Correspondence: whuang@age.mpg.de; aantebi@age.mpg.de

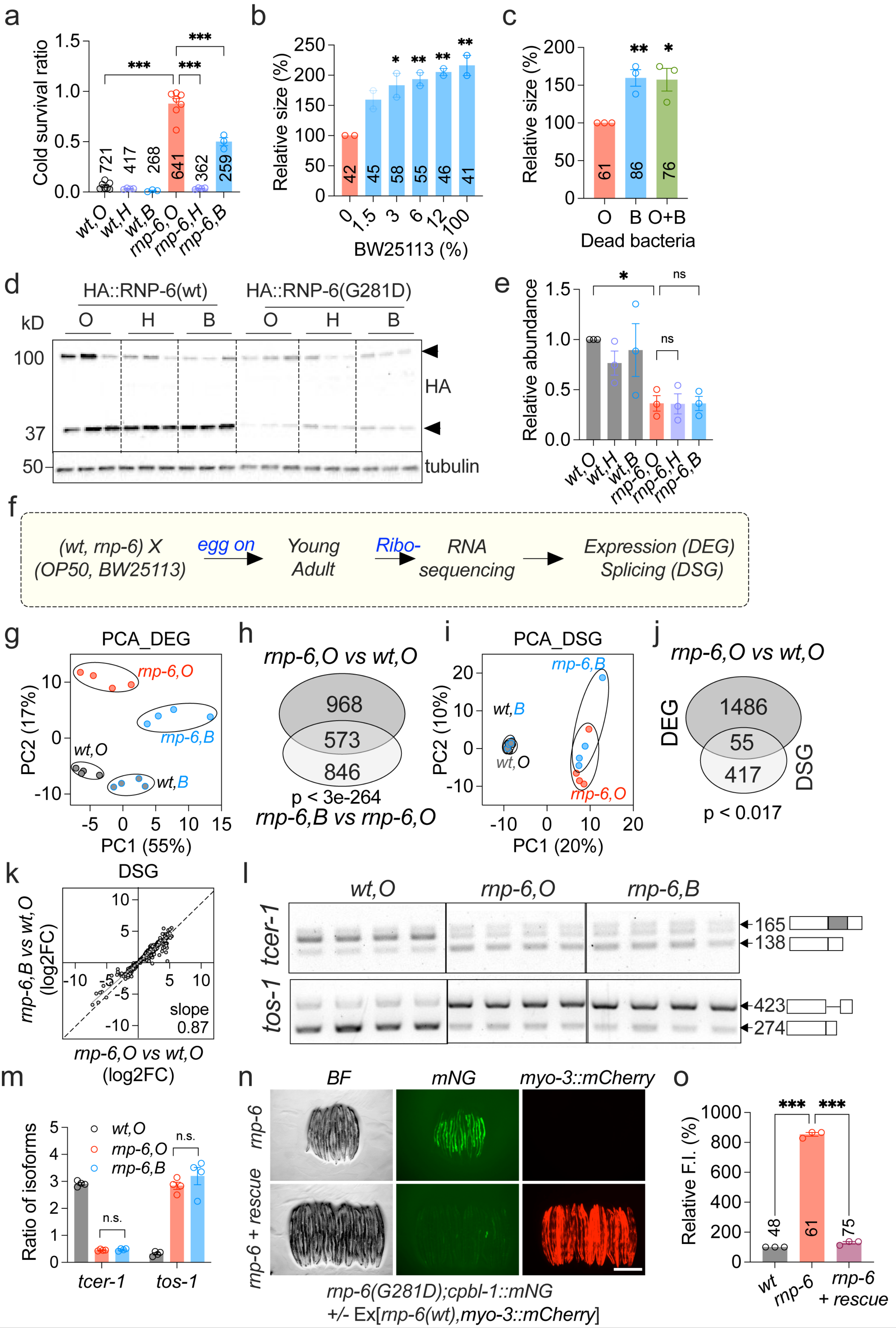
a
Cold survival ratio
wt,O
wt,H
wt,B
rnp-6,O
rnp-6,H
rnp-6,B
721 417 268 641 362 259
***
b
Relative size (%)
BW25113 (%)
0 1.5 3 6 12 100
42 45 58 55 46 41
c
Relative size (%)
O B O+B
Dead bacteria
61 86 76
d
HA::RNP-6(wt)
HA::RNP-6(G281D)
kD
O H B
100
37
50
HA
tubulin
e
Relative abundance
ns
f
(wt, rnp-6) X (OP50, BW25113)
egg on
Young Adult
Ribo-
RNA sequencing
Expression (DEG)
Splicing (DSG)
g
PCA_DEG
PC2 (17%)
PC1 (55%)
h
rnp-6,O vs wt,O
968
573
846
p < 3e-264
rnp-6,B vs rnp-6,O
i
PCA_DSG
PC2 (10%)
PC1 (20%)
j
rnp-6,O vs wt,O
DEG
DSG
1486
55
417
p < 0.017
k
DSG
rnp-6,B vs wt,O (log2FC)
rnp-6,O vs wt,O (log2FC)
slope 0.87
l
tcer-1
tos-1
165
138
423
274
m
Ratio of isoforms
n.s.
n
BF
mNG
myo-3::mCherry
rnp-6
rnp-6 + rescue
rnp-6(G281D);cpbl-1::mNG
+/- Ex[rnp-6(wt),myo-3::mCherry]
o
Relative F.I. (%)
wt
rnp-6
rnp-6 + rescue
48 61 75

Supplementary Figure 1. A bacterial *E. coli* K12 diet mitigates developmental defects in an *rnp-6*/VRJS *C. elegans* model. a, Assessment of cold tolerance in worms fed *E. coli* K12 strains; bars represent the ratio of alive to total worms per genotype and diet after cold shock (*n* = 3–7). **b**, Evaluation of BW25113 dilution effects on worm body size (*n* = 2). **c**, Body size of *rnp-6* mutant worms fed UV-killed BW25113 and OP50-BW25113 (ratio 1:1) mixture (*n* = 3). **d–e**, Total RNP-6 protein abundance in response to K12 diets. The total amounts of two predominant RNP-6 isoforms (as denoted by arrowheads) were quantified (*n* = 3). **f**, Overview of RNA-seq experimental design. Ribo-, ribosomal RNA depletion. **g**, Principal component analysis of differentially expressed genes (DEG), four biological replicates were analyzed for each condition. **h**, Venn diagram of DEGs changed by *rnp-6* mutation on OP50 and BW25113. **i**, Principal component analysis of differentially spliced genes (DSG). **j**, Overlap between DEGs and DSGs, shown by Venn diagram. **k**, Effect of BW25113 on alternative splicing; 638 events significantly altered by *rnp-6(G281D)* on OP50 are displayed. **l–m**, RT-PCR splicing analyses of *tos-1* and *tcer-1* (*n* = 4). Y-axis (**m**) indicates ratio of bigger isoform to smaller isoform. **n–o**, Effect of *rnp-6(wt)* overexpression on CPBL-1::mNG reporter expression. *myo-3::mCherry* co-injection marker indicates transgenic animals (*n* = 3, scale bar: 500 µm). Each open circle in a bar graph represents the average value of multiple worms in one experimental replicate. Data are presented as mean ± s.e.m. of averages unless otherwise indicated. "*n*" denotes experimental replicates, with animal counts summarized in each bar graph. Statistical significance was determined using one-way ANOVA with Dunnett's multiple comparisons (**a**, **b**, **c**, **e**, **m**, **o**). Source data are provided as a Source Data file.

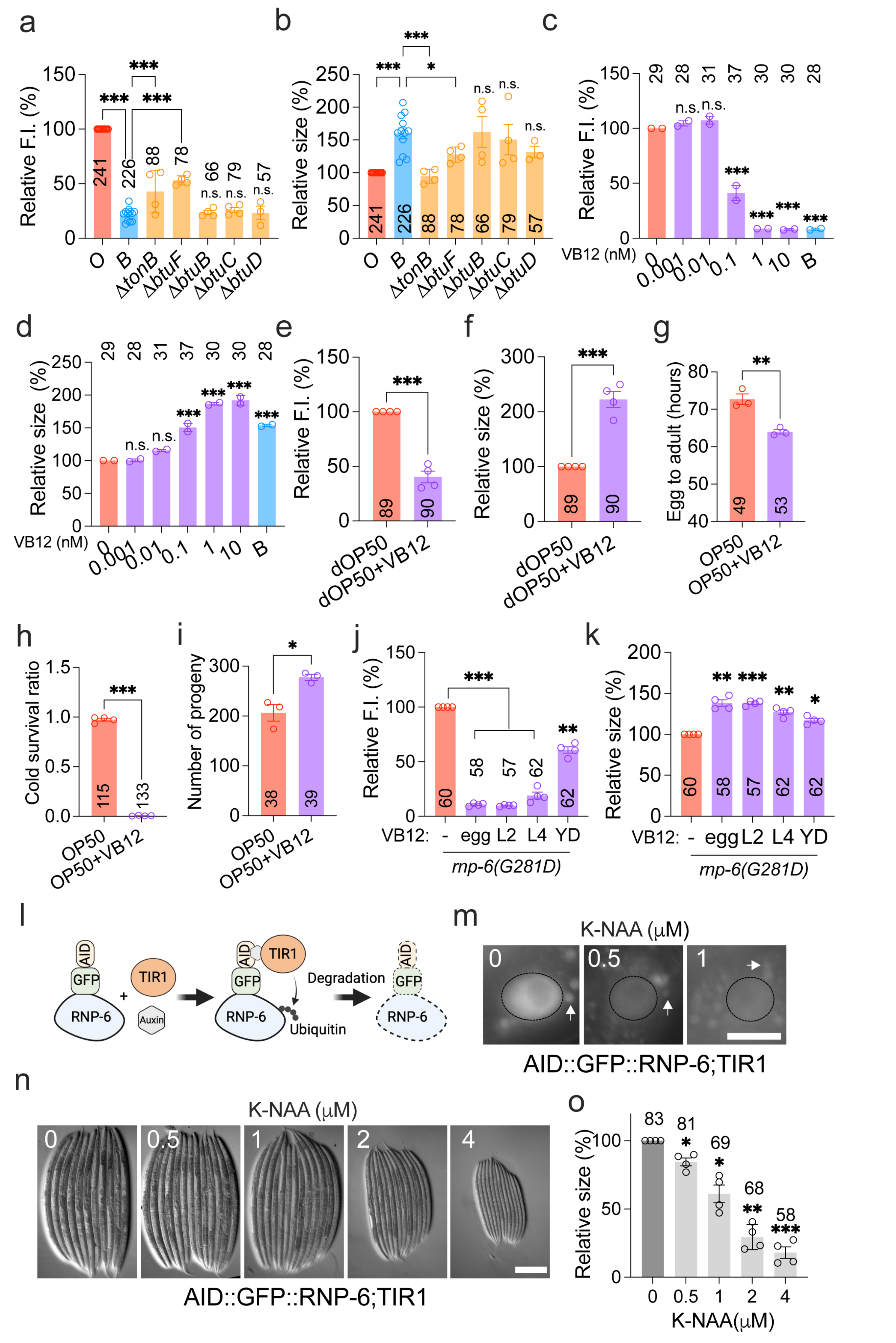
a
Relative F.I. (%)
150
100
50
0
***
***
***
241
226
88
78
66
79
57
n.s.
n.s.
n.s.
O
B
ΔtonB
ΔbtuF
ΔbtuB
ΔbtuC
ΔbtuD
b
Relative size (%)
250
200
150
100
50
0
***
***
*
n.s.
n.s.
n.s.
241
226
88
78
66
79
57
O
B
ΔtonB
ΔbtuF
ΔbtuB
ΔbtuC
ΔbtuD
c
Relative F.I. (%)
150
100
50
0
29
28
31
37
30
30
28
n.s.
n.s.
***
***
***
***
VB12 (nM)
0
0.001
0.01
0.1
1
10
B
d
Relative size (%)
250
200
150
100
50
0
29
28
31
37
30
30
28
n.s.
n.s.
***
***
***
***
VB12 (nM)
0
0.001
0.01
0.1
1
10
B
e
Relative F.I. (%)
150
100
50
0
***
89
90
dOP50
dOP50+VB12
f
Relative size (%)
300
200
100
0
***
89
90
dOP50
dOP50+VB12
g
Egg to adult (hours)
80
70
60
50
40
**
49
53
OP50
OP50+VB12
h
Cold survival ratio
1.5
1.0
0.5
0.0
***
115
133
OP50
OP50+VB12
i
Number of progeny
300
200
100
0
*
38
39
OP50
OP50+VB12
j
Relative F.I. (%)
150
100
50
0
***
**
60
58
57
62
62
VB12:
-
egg
L2
L4
YD
rnp-6(G281D)
k
Relative size (%)
200
150
100
50
0
**
***
**
*
60
58
57
62
62
VB12:
-
egg
L2
L4
YD
rnp-6(G281D)
l
AID
GFP
RNP-6
TIR1
Auxin
Degradation
Ubiquitin
m
K-NAA (μM)
0
0.5
1
AID::GFP::RNP-6;TIR1
n
K-NAA (μM)
0
0.5
1
2
4
AID::GFP::RNP-6;TIR1
o
Relative size (%)
100
50
0
83
81
69
68
58
*
*
**
***
0
0.5
1
2
4
K-NAA(μM)

Supplementary Figure 2. A complementary two-way genetic screen identifies vitamin B_{12} as an alleviator of *rnp-6(G281D)* mutant defects. **a–b**, Effects of bacterial vitamin B_{12} transporter knockout on *cpbl-1* reporter expression (**a**) and body size (**b**) in *rnp-6(G281D)* mutants (*n* = 4–12). **c–d**, Dose-response effect of vitamin VB12 supplementation on *rnp-6(G281D)* phenotypes (*n* = 2). **e–f**, Impact of VB12 supplementation on *rnp-6(G281D)* mutants fed UV-killed OP50 (*n* = 4). **g–i**, Effects of VB12 supplementation on developmental rate (**g**, *n* = 3), cold tolerance (**h**, *n* = 4), and brood size (**i**, *n* = 3). **j–k**, Effects of VB12 supplementation administered from distinct developmental stages on *cpbl-1* reporter expression (**j**) and body size (**k**) in *rnp-6(G281D)* mutants (*n* = 4). YD, young adult stage. Worms were imaged on day 2 of adulthood. **l**, Schematic illustrating the RNP-6 inducible degradation system. **m**, Effects of K-NAA supplementation on GFP::RNP-6 fluorescence intensity on OP50 (scale bar: 10 µm). The nuclei of the intestine are indicated with circles. The arrows indicate gut autofluorescent granules. **n–o**, Impact of various K-NAA concentrations on body size on OP50 (*n* = 4, scale bar: 200 µm). Final concentration of VB12 is 1 nM unless otherwise noted. Each open circle in a bar graph represents the average value of multiple worms in one experimental replicate. Data are presented as mean ± s.e.m. of averages unless otherwise indicated. "*n*" denotes experimental replicates, with animal counts summarized in each bar graph. Statistical significance was determined using one-way ANOVA with Dunnett's multiple comparisons. Significance is represented as $^{*}P < 0.05$, $^{**}P < 0.01$, $^{***}P < 0.001$, n.s.: not significant. Schematic (**l**) was generated with BioRender. Source data are provided as a Source Data file.

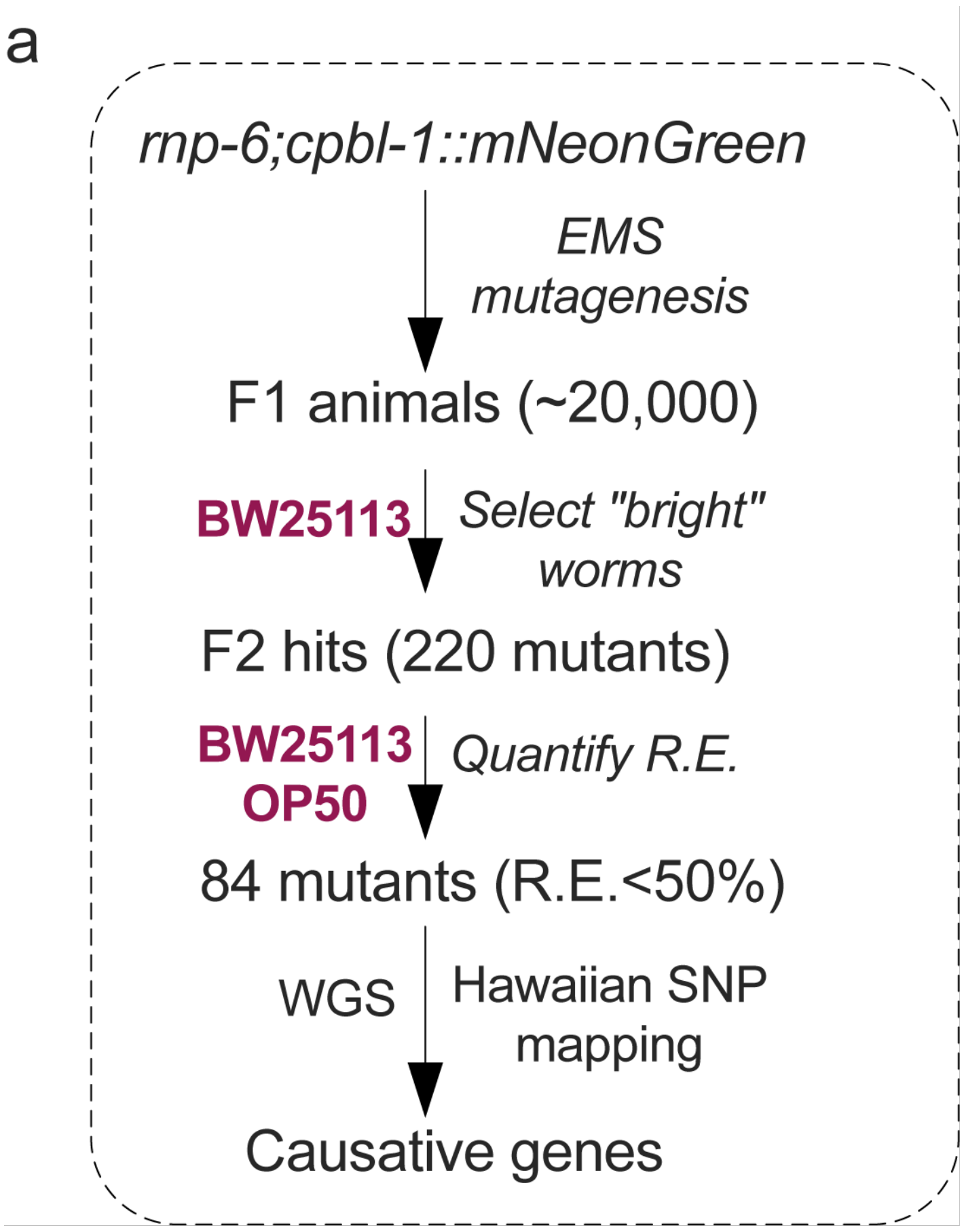

Supplementary Figure 3. Vitamin B_{12} alleviates *rnp-6(G281D)* mutant defects via methionine metabolism pathways. a, Schematic of EMS mutagenesis screen for host factors. R.E. (rescue efficacy) = (Fluorescent intensity on OP50 - Fluorescent intensity on BW25113)/ Fluorescent intensity on OP50).

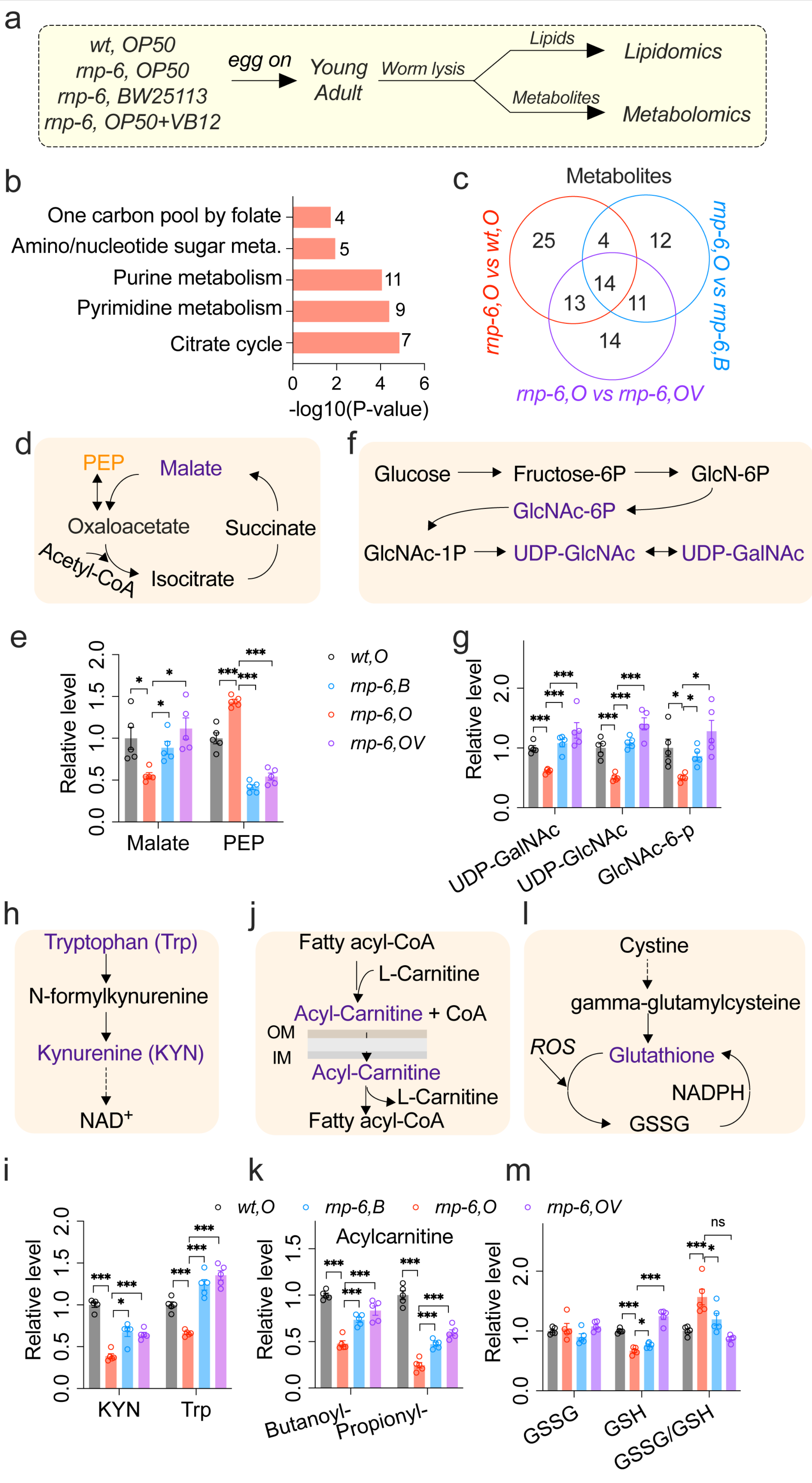
a
wt, OP50
rnp-6, OP50
rnp-6, BW25113
rnp-6, OP50+VB12
egg on
Young Adult
Worm lysis
Lipids
Lipidomics
Metabolites
Metabolomics
b
One carbon pool by folate
Amino/nucleotide sugar meta.
Purine metabolism
Pyrimidine metabolism
Citrate cycle
-log10(P-value)
c
Metabolites
rnp-6,O vs wt,O
rnp-6,O vs rnp-6,B
rnp-6,O vs rnp-6,OV
d
PEP
Malate
Oxaloacetate
Succinate
Acetyl-CoA
Isocitrate
f
Glucose
Fructose-6P
GlcN-6P
GlcNAc-6P
GlcNAc-1P
UDP-GlcNAc
UDP-GalNAc
e
Relative level
Malate
PEP
wt,O
rnp-6,B
rnp-6,O
rnp-6,OV
g
Relative level
UDP-GalNAc
UDP-GlcNAc
GlcNAc-6-p
h
Tryptophan (Trp)
N-formylkynurenine
Kynurenine (KYN)
NAD+
j
Fatty acyl-CoA
L-Carnitine
Acyl-Carnitine + CoA
OM
IM
Acyl-Carnitine
L-Carnitine
Fatty acyl-CoA
l
Cystine
gamma-glutamylcysteine
ROS
Glutathione
NADPH
GSSG
i
Relative level
KYN
Trp
k
Acylcarnitine
Relative level
Butanoyl-
Propionyl-
m
Relative level
GSSG
GSH
GSSG/GSH
ns

Supplementary Figure 4. Vitamin B_{12} restores methylation potential and phosphatidylcholine metabolism in *rnp-6* mutants. a, Workflow for metabolomic and lipidomic analyses. Five replicates per condition. **b**, MetaboAnalyst-based pathway analysis of metabolites significantly altered in *rnp-6(G281D)* mutants. **c**, Venn diagram of significantly altered metabolites. Simplified schematics and abundance changes are shown for the following pathways: **d–e**, tricarboxylic acid (TCA) cycle; **f–g**, amino sugar and nucleotide sugar metabolism; **h–i**, kynurenine pathway; **j–k**, mitochondrial fatty acid metabolism; **l–m**, glutathione metabolism. Each open circle in a bar graph represents the average value of multiple worms in one experimental replicate. Data are presented as mean ± s.e.m. of averages unless otherwise indicated. "*n*" denotes experimental replicates, with animal counts summarized in each bar graph. Statistical significance was determined using one-way ANOVA with Dunnett's multiple comparisons. Significance is represented as $^*P < 0.05$, $^{**}P < 0.01$, $^{***}P < 0.001$, n.s.: not significant. Source data are provided as a Source Data file.

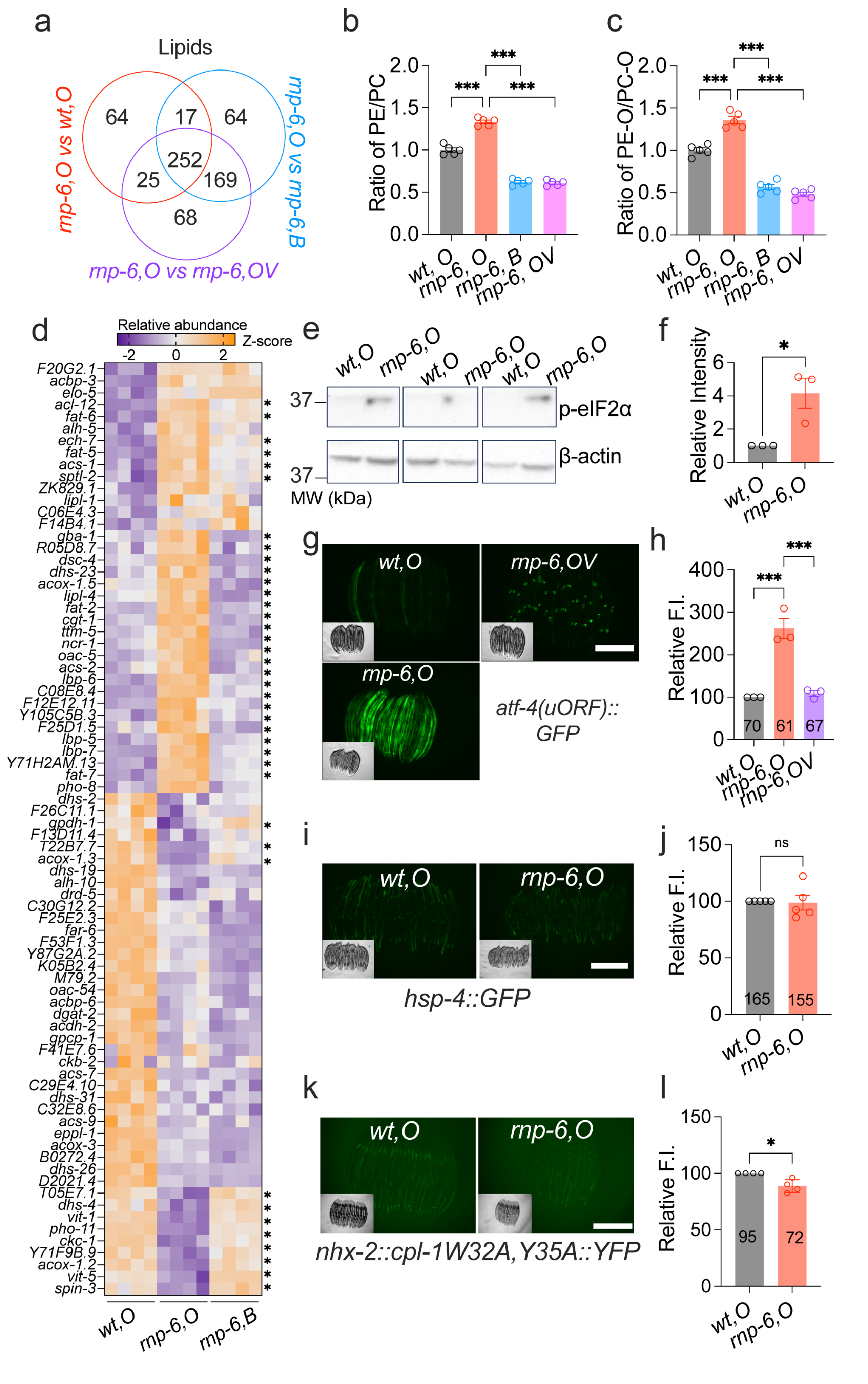
a
Lipids
rnp-6,O vs wt,O
rnp-6,O vs rnp-6,B
rnp-6,O vs rnp-6,OV
64
17
64
252
25
169
68
b
Ratio of PE/PC
wt, O
rnp-6, O
rnp-6, B
rnp-6, OV
c
Ratio of PE-O/PC-O
d
Relative abundance
Z-score
wt,O
rnp-6,O
rnp-6,B
e
wt,O
rnp-6,O
p-eIF2α
β-actin
MW (kDa)
37
f
Relative Intensity
g
wt,O
rnp-6,OV
rnp-6,O
atf-4(uORF)::GFP
h
Relative F.I.
70
61
67
i
hsp-4::GFP
j
ns
165
155
k
nhx-2::cpl-1W32A,Y35A::YFP
l
95
72

Supplementary Figure 5. Vitamin B_{12} restores phosphatidylcholine metabolism and the integrated stress response in *rnp-6* mutants. a, Venn diagram of significantly altered lipid species. **b**, Ratio of phosphatidylethanolamine (PE) to phosphatidylcholine (PC) changes. **c**, Ratio of ether-linked PE (PE-O) to ether-linked PC (PC-O) changes. **d**, Heat map of differentially expressed genes (DEGs) associated with lipid metabolism; "*" denotes genes significantly restored by BW25113 diet. **e–f**, Western blot analysis of eIF2α phosphorylation in *rnp-6* mutants, with three biological replicates analyzed (n = 3). **g–h**, Representative images and quantification of ISR reporter strain *Patf-4(uORF)::GFP* expression (scale bar: 500 µm). **i–j**, Representative images and quantification of unfolded protein response of the endoplasmic reticulum (UPR^{ER}) reporter strain *hsp-4::GFP* expression (n = 5, scale bar: 500 µm). **k–l**, Representative images and quantification of CPL-1 reporter strain *cpl-1W32A,Y35A::YFP* expression (*n* = 4, scale bar: 500 µm). Each open circle in a bar graph represents the average value of multiple worms in one experimental replicate. Data are presented as mean ± s.e.m. of averages unless otherwise indicated. "*n*" denotes experimental replicates, with animal counts summarized in each bar graph. Statistical significance was determined using unpaired t-test (**f**, **j**, **l**) and one-way ANOVA with Dunnett's multiple comparisons (**h**). Significance is represented as *P < 0.05, **P < 0.01, ***P < 0.001, n.s.: not significant. Source data are provided as a Source Data file.

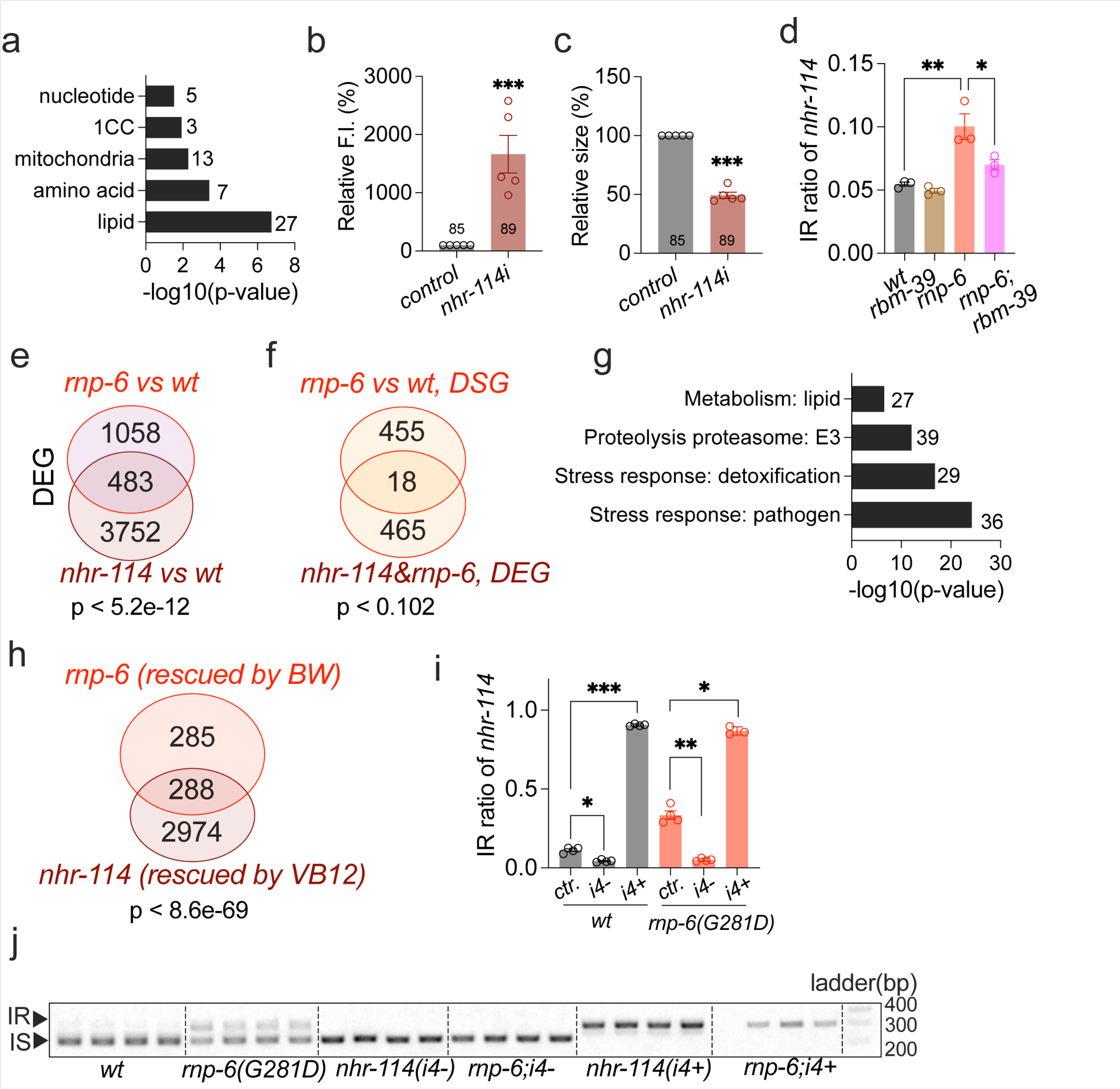

Supplementary Figure 6. Aberrant splicing of transcription factor *nhr-114* drives *rnp-6* mutant defects. a, Gene set enrichment analysis of differentially spliced genes involved in metabolism, performed with WormCat 2.0. **b–c**, Quantification of CPBL-1::mNG fluorescence intensity and worm body size after *nhr-114* RNAi (n = 5) treatment. **d**, Quantification of *nhr-114* intron 4 retention in *rbm-39(S294L)* mutants from RNAseq data. n=3. **e**, Venn diagram illustrating differentially expressed genes in the *nhr-114* deletion mutant and the *rnp-6* mutant. **f**, Venn diagram illustrating genes that are differentially spliced. **g**, WormCat gene set enrichment analysis of the overlapped genes in (e). **h**, Venn diagram of differentially expressed genes significantly rescued by either VB12 supplementation in *nhr-114* deletion mutants or BW25113 diet in *rnp-6* mutants. **i–j**, RT-PCR analysis of *nhr-114* intron 4 retention in different *nhr-114* gene editing mutants. *nhr-114(+)* results in almost complete intron retention; one biological replicate for *rnp-6; nhr-114(i4+)* was excluded from quantification due to technical failure (n = 3–4). Each open circle in a bar graph represents the average value of multiple worms in one experimental replicate. Data are presented as mean ± s.e.m. of averages unless otherwise indicated. "*n*" denotes experimental replicates, with animal counts summarized in

each bar graph. Statistical significance was determined using unpaired t-test (**b, c**) and one-way ANOVA with Dunnett's multiple comparisons (**d, i**). Significance is represented as *P < 0.05, **P < 0.01, ***P < 0.001, n.s.: not significant. Source data are provided as a Source Data file.

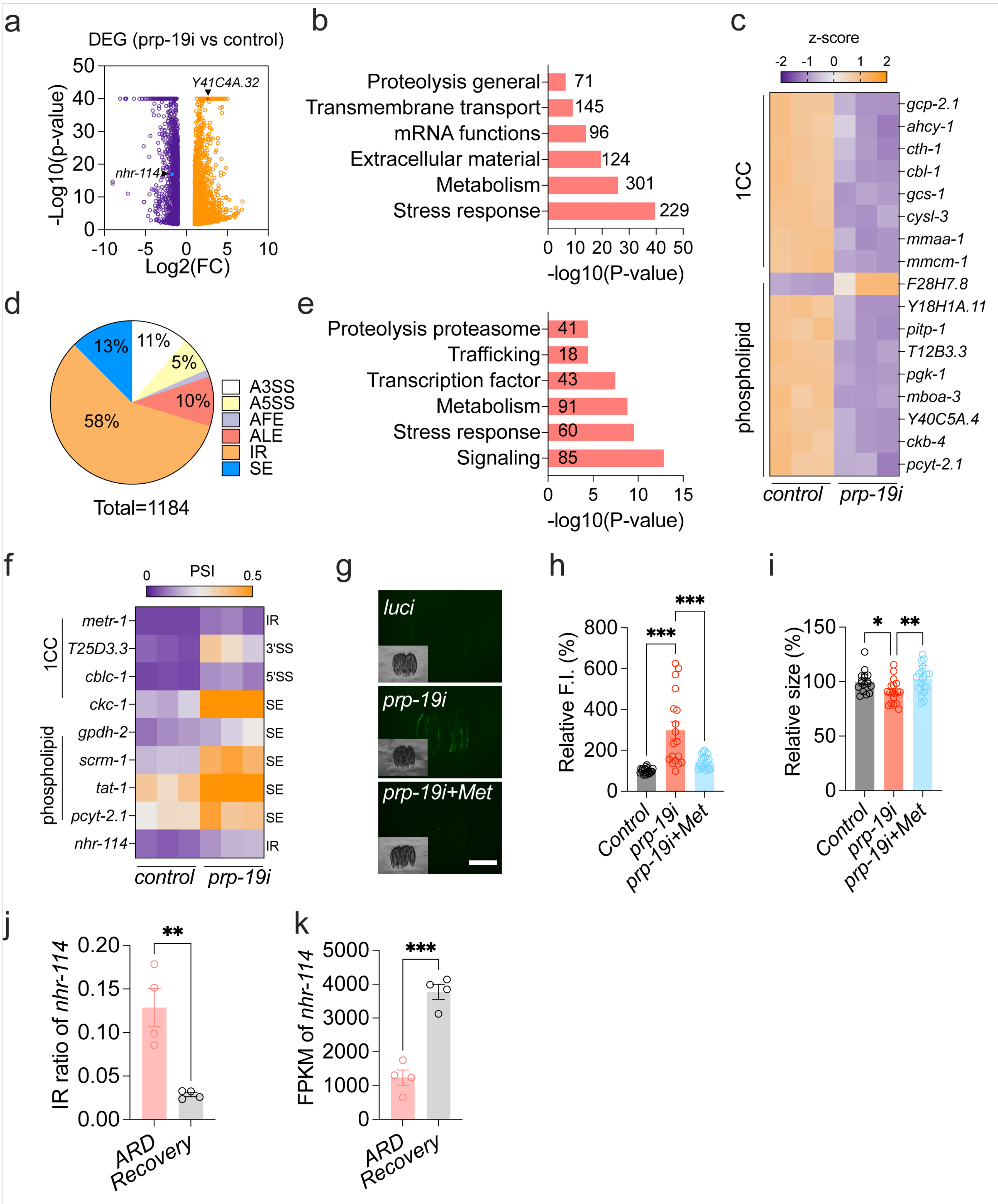

Supplementary Figure 7. *prp-19* inhibition dysregulates metabolism. a, Volcano plot of differentially expressed genes caused by *prp-19* RNAi. Black arrowheads indicate *cpbl-1* and *nhr-114*, respectively. **b**, Wormcat enrichment analysis of differentially expressed genes. **c**, Heatmap of differentially expressed genes related to 1C and phospholipid metabolism. **d**, Pie chart of differentially spliced genes caused by *prp-19* RNAi. **e**, Wormcat enrichment analysis of differentially spliced genes. **f**, Heatmap of differentially spliced genes related to 1C and phospholipid metabolism. **g**, Representative images of CPBL-1::mNG upon *prp-19* RNAi treatment (scale bar: 500 μm). **h**, Quantification of CPBL-1::mNG fluorescent intensity upon *prp-19*

RNAi treatment. **i**, Quantification of body size upon *prp-19* RNAi treatment. j-**k,** Quantification of *nhr-114* intron 4 retention and expression levels in ARD worms, analyzed by RNA-seq (n = 4). Statistical significance was determined using unpaired t-test (**j, k**) and one-way ANOVA with Dunnett's multiple comparisons (**h, i**). Significance is represented as *P < 0.05, **P < 0.01, ***P < 0.001, n.s.: not significant. Source data are provided as a Source Data file.

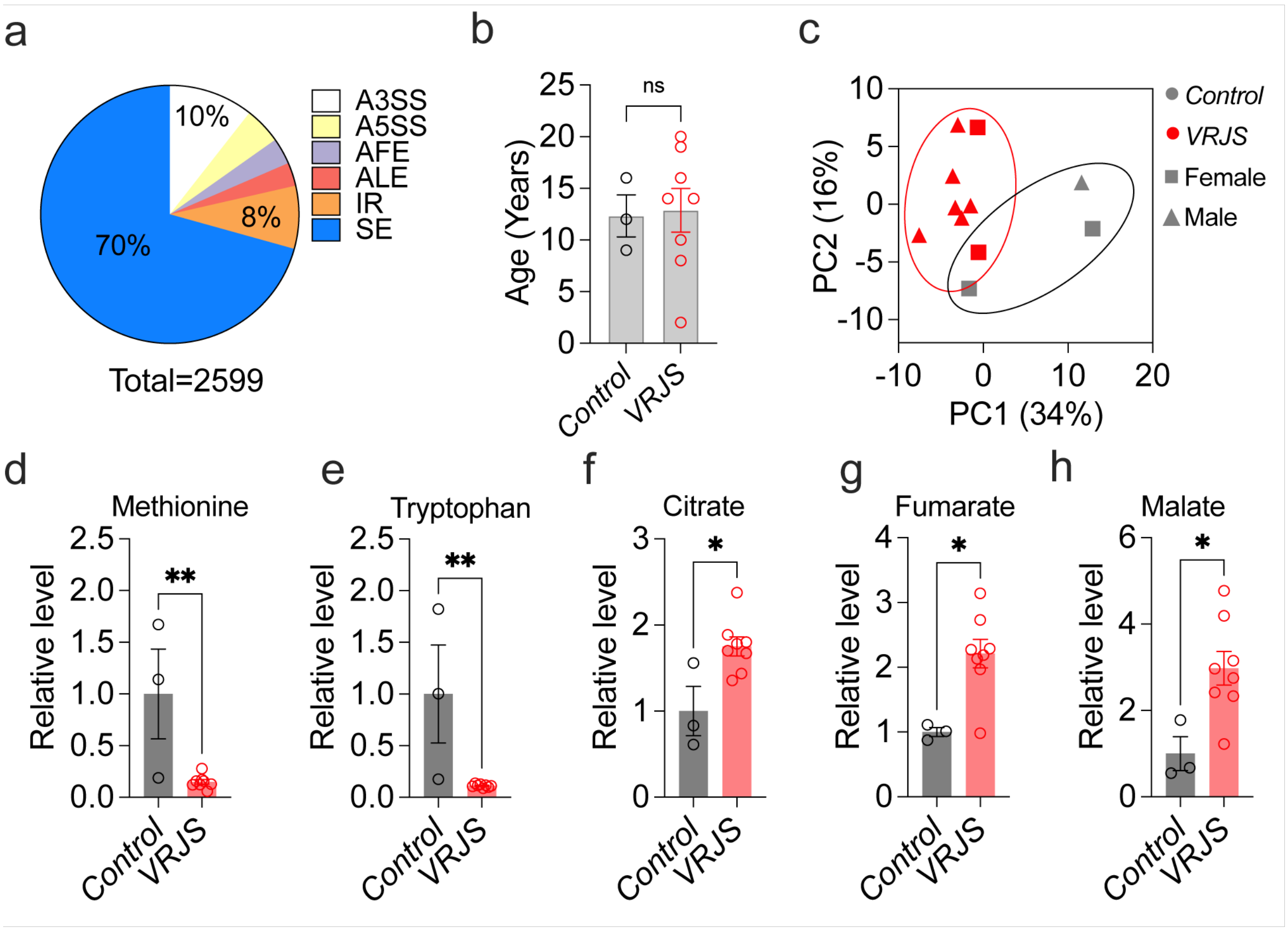

Supplementary Figure 8. PUF60 deficiency impairs one-carbon and phospholipid metabolism in human cells. a, Pie chart displaying differentially spliced events in the siPUF60-treated PC9 cell line. **b**, Age distribution of control and VRJS patient plasma samples. **c**, PCA analysis of metabolomic and lipidomic data. **d-h**, The relative abundance of methionine (d), tryptophan (e), citrate (f), fumarate (g) and malate (h) in VRJS patient plasma. Statistical significance was determined using unpaired t-test. Significance is represented as *P < 0.05, **P < 0.01, ***P < 0.001, n.s.: not significant. Source data are provided as a Source Data file.

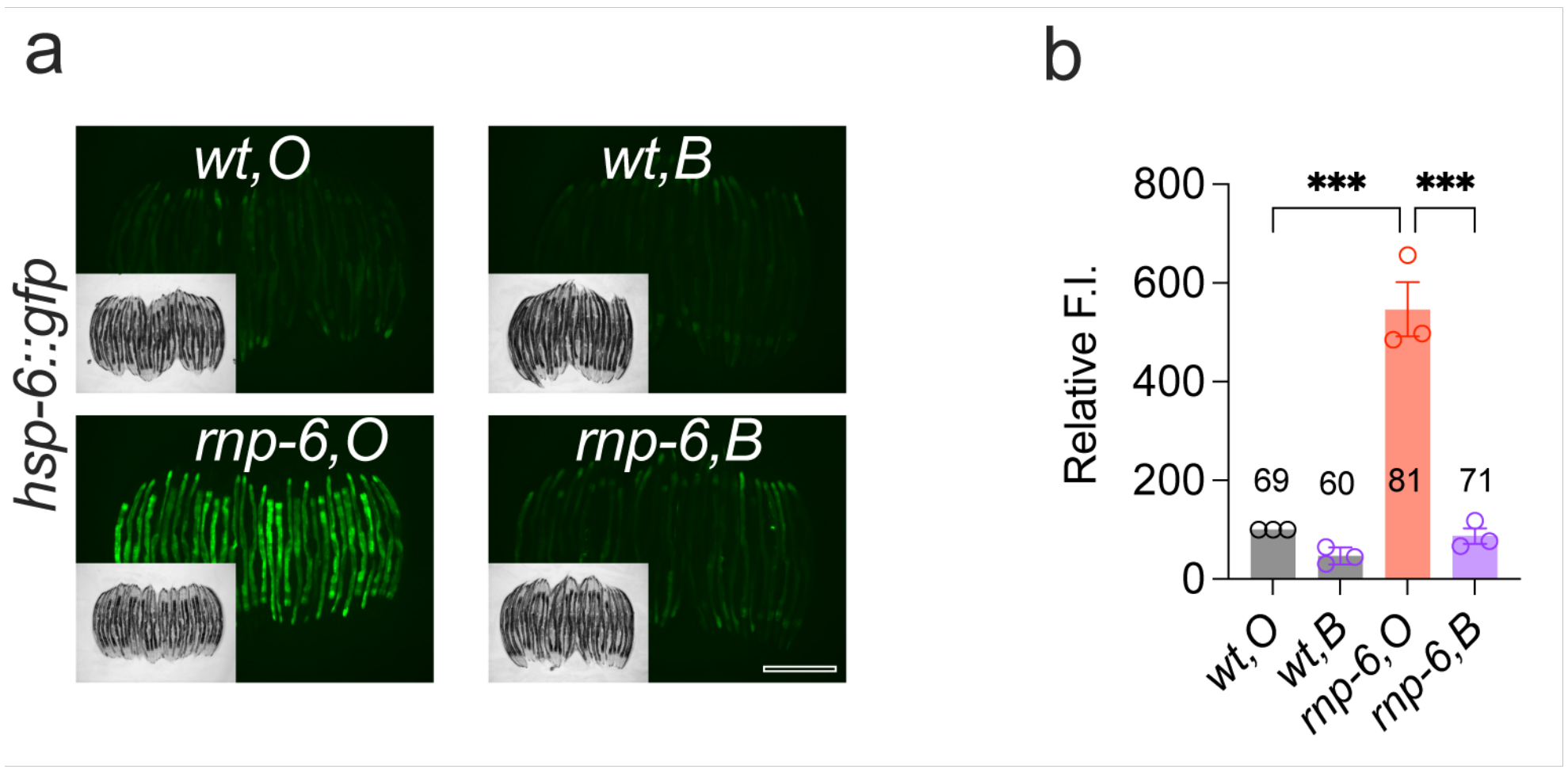

Supplementary Figure 9. ***E. coli*** **BW25113 alleviates mitochondrial stress in** ***rnp-6(G281D)*** **mutants. a,** Representative images of *hsp-6::gfp* reporter expression in *rnp-6* mutants ($n = 3$, scale bar: 500 µm). **b**, Quantification of *hsp-6::gfp* reporter fluorescent intensity. Each open circle in a bar graph represents the average value of multiple worms in one experimental replicate. Data are presented as mean ± s.e.m. of averages unless otherwise indicated. "*n*" denotes experimental replicates, with animal counts summarized in each bar graph. One-way ANOVA with Dunnett's multiple comparison test was used for statistical analysis. Significance is represented as ***$P < 0.001$. Source data are provided as a Source Data file.
