# Supplementary material for: Vitamin B12 alleviates spliceosomopathy via phospholipid remodeling
Source: Nat Commun. 2026 Aug 7;17:8011. doi: 10.1038/s41467-026-76295-9 (PMC13451374; doi:10.1038/s41467-026-76295-9)
Supplement: Supplementary file 2 [file 41467_2026_76295_MOESM2_ESM.pdf]

nature portfolio

Peer Review File

Vitamin B_{12} alleviates spliceosomopathy via phospholipid remodeling

Corresponding Author: Professor Adam Antebi

This file contains all reviewer reports in order by version, followed by all author rebuttals in order by version.

Version 0:

Reviewer comments:

Reviewer #1

(Remarks to the Author)
Here, Huang et al. present a really thorough investigation of how RNP-6 regulates the expression of genes involved in S-adenosylmethionine and S-adenosylhomocysteine cycling and metabolism. In addition, the authors present the surprising findings that increased dietary B12 supplementation can alleviate many of the defects observed in their C. elegans rnp-6 mutant model. As part of these studies the authors conduct two independent and unbiased genetic screens, one in E. coli (C. elegans food source), and one in C. elegans itself. In both cases the authors identify genes that converge on vitamin B12 and methionine/SAM metabolism. These findings, coupled with substantial additional analyses and validation, make a convincing case that specific metabolic defects underlie many of the organismal level pathologies in rnp-6 mutant animals. The authors further build on these findings and demonstrate that they are not unique to rnp-6 mutant animals but that similar observations were observed for mutations in other splicing factors (prp-19) and thus were not specific to the rnp-6 mutant model. They also add some data that suggests, even if not conclusive, that human patient cell line models and humans with Verheij syndrome might have similar metabolic defects. Overall, I think this is a very well done study with substantial data. I think the data supports the claims made in the manuscript and its well written. As is clear in the model in Figure 7 some questions remain, however I think there is already substantial data in this manuscript as is and that the conclusions are strong and represent a significant advance in the field. Given the surprising and very interesting findings and the already rigorous approach. I do not feel that additional experiments are required for publication, which is not something I've been able to do often on a first review. Interesting work and very well done.

Nick Burton

Reviewer #2

(Remarks to the Author)
Comments on Figure 5
Wenming Huang et al Fig. 5 presents a coherent and logically structured set of experiments linking rnp-6–dependent splicing defects to functional loss of the transcription factor nhr-114. The progression from global splicing alterations to a specific causal intron retention event is well motivated, and the genetic rescue experiments provide strong support for the central conclusion that aberrant nhr-114 splicing is a major driver of rnp-6 mutant phenotypes. Overall, Figure 5 is a strong centerpiece of the manuscript and provides compelling genetic evidence linking spliceosomal dysfunction to metabolic pathology via nhr-114 mis-splicing.

Major points:
1. Scope of causality
As I understand nhr-114 promotes a change in splicing that is associated with the observed phenotype. The authors cherry pick one event to better connect the two. While the specific event chosen is well demonstrated as a target of nhr-114, some of the other targets can be non-direct. For this reason I would edit the text of "primarily caused" to something more subtle.

2. Fig. 1F shows a sashimi plot of the intron, one can also absorve that the intron upstream is also retained in response to nhr-114. This should be discussed.
Does crispr in panel k predicted to affect the retention of this intron?

3. Protein-level validation
The reduction in GFP::NHR-114 signal (panels i–j) supports functional loss, but it is not entirely clear whether decreased fluorescence reflects reduced protein abundance, altered stability, or cell-type–specific effects. A brief clarification in the legend or text noting whether GFP tagging preserves normal localization and function would improve interpretability.
Minor comments:
Many of the acronyms are not explained or explained many times: Verheij syndrome (OMIM #615583) VRJS, Vitamin B12, DSGs

Reviewer #3

(Remarks to the Author)
In the manuscript, Huang et al. analyzed rnp-6(G281D) hypomorphic mutant of C. elegans and demonstrate that dysregulation of this splicing factor causes smaller body size and slower growth rate through imbalance in SAM and phosphatidylcholine metabolism.
First, they discovered that the phenotypes were restored by feeding with distinct E. coli strains and identified hundreds of differentially expressed and/or spliced genes on diet. They established a Y41C4A.32 reporter to read out the rnp-6(G281D)/OP50-specific condition and identified vitamin B12 (VB12) as sufficient to restore the phenotypes except for longevity. They also searched for host factors and identified methionine metabolism pathways, consistent with VB12 requirement. Metabolomic and lipodomic analyses revealed imbalances in SAM/SAH and phosphatidylcholine, and VB12 supplementation restored most of the lipid metabolism. They further screened direct RNP-6 targets and identified intron retention of nhr-114 as a primary cause of the rnp-6(G281D) phenotypes. They further demonstrate that mTORC signaling is affected in the mutant and is critical for phenotype restoration by VB12 supplementation. Mutations in the human ortholog of rnp-6, PUF60, is know to cause Verheij syndrome (VRJS) and the authors demonstrate that knockdown of PUF60 also affected splicing of a similar set of metabolism-related genes. Furthermore, metabolites and lipids are affected in VRJS patient bloods.
From these and other results the authors propose that spliceosomopathies, in both C. elegans and humans, can be alleviated with VB12.

Overall, the study is well organized, and the results are clearly described. The main conclusion of the study is well supported by the key experiments such as genome editing of the nhr-114 gene and use of the raga-1 (lof) mutant. I have a few concerns regarding the description in the main text.

Major criticisms:
1. Lines 195-196, “We found that deletion of sams-1 fully abolished the rescue by BW25113, VB12 and methionine in rnp-6(G281D) mutants (Figure 3h–i)”: Figures 3h and 3j demonstrate that expression of the Y41C4A.32 reporter is suppressed by the sams-1 mutation in the rnp-6(G281D) mutant, and that feeding with BW25113 or supplementation with VB12 or methionine enhances its expression. If enough amount of SAM is critical for suppressing the reporter in the rnp-6(G281D) mutant, its expression would be higher in the sams-1 background fed with OP50. Please discuss this unexpected result.
2. Lines 201-203, “These findings support a model in which SAM-dependent phosphatidylcholine synthesis constitutes a major downstream mechanism by which K12-type E. coli and VB12 rescue rnp-6 mutant phenotypes.”: Even though choline supplementation bypassed the requirement for sams-1 in the rnp-6(G281D) mutants (Figure 3j–k), the results does not necessarily indicate that phosphatidylcholine is the critical downstream mechanism for rescue as in the title; choline supplementation may have just attenuated consumption of SAM.
3. Lines 305-306, “~60% (288/483) of the shared genes were rescued by VB12 supplementation in the nhr-114 mutant and by BW25113 feeding in the rnp-6 mutant (Figure S6h, Table S10)”: Please check if this description is correct. I understand there are 573 genes rescued by BW25113 feeding in the rnp-6 mutant (Figure S1h). The source of the RNA-seq data for the nhr-114 mutant supplemented with VB12 is not mentioned.

Minor points:
1. Lines 104-105, “Feeding with BW25113 significantly changed the expression of 1319 genes in rnp-6 mutants”: It seems from Figure S1h that there are 1419 DEGs.
2. Lines 105-107, “WormCat 2.0 gene set analysis of the BW25113-restored genes revealed significant enrichment in ... (Figure 1f).”: The main text mentions analysis on 573 of “BW25113-restored” genes whereas Figure 1f analyzes 1319 of “BW25113-responsive” genes. Please clarify which is correct.
3. Line 264, “most SE events (217 out of 227 events) decreased”: It is confusing whether exon skipping was decreased or exon inclusion was decreased.
4. Line 284, “nhr-114 showed the strongest effect on both phenotypes (Figure 5d–e, S6b–d)”: Figure S6d may not be related to this claim.
5. Lines 297-298, “483 of these genes were also significantly altered in rnp-6(G281D) (overlap significance, $p < 5e-12$) (Figure S6e–f; Table S10).”: Figure S6f may not be related to this claim.
6. Lines 303-304, “Less than 4% (18/483) of the overlapped differentially expressed genes displayed aberrant splicing in rnp-6 mutants (overlap significance, $p < 0.102$) (Table S10)”: Figure S6f may also support this claim.
7. Line 313, “nhr-114(i4+) phenocopied rnp-6(G281D)”: Please clarify if this i4+ knock-in allele is homozygous or heterozygous.
8. Lines 314-315, “while not further elevating Y41C4A.32 expression in rnp-6(G281D) mutants (Figure 5m–o)”: It seems from Figure 5l-m that Y41C4A.32 expression is elevated in rnp-6(G281D) mutants. I think this makes sense if the i4+ allele is

homozygous. Figure 5l is not cited in the main text.
9. Lines 346-347, "Under ARD conditions nhr-114 intron 4 retention was significantly increased by ~10% relative to fed controls (Figure S7k) accompanied by reduced levels of nhr-114 mRNA (Figure S7l).": These claims should cite Figure S7j and S7k, respectively. Figures S7j-k compare ARD conditions with Recovery conditions and lack normal conditions.
10. Lines 393-394, "Although the levels of PC remained unchanged, the levels of PE and the ratio of PC/PE were slightly increased (Figure 7d).": Figure 7d demonstrates that the level of PE was slightly decreased.
11. Figure 1b-c, g and many others: Please make it clear in the legend that each open circle represent the average of multiple worms in a set of experiment. "Data are presented as mean ±s.e.m.": Please clarify whether the mean and the s.e.m. are those of all worms or of the averages.
12. Legend to Figure 1g, "F.C., fold change": Not involved in the Figure.
13. Figure 2d: Not cited in the main text.
14. Figure 3f-g and j-k: Please clarify that these worms were fed with OP50.
15. Legend to Figure 6c, "Scale bar, 20 mm.": This may be micrometer.
16. Figure S1, lines 4-5, "bars represent the ratio of alive to dead worms": Methods section describes "Cold survival ratio was measured as the ratio of the number of live worms to the number of total worms." Please make sure which is correct.
17. Figure S1, line 5, "diet after cold shock": It seems from the Method section that the E. coli diet was NOT used after cold shock.
18. Figure S1m: Unclear what the y-axis indicates.
19. Figure S2j-k: Please explain what "YD" indicates.
20. Figure S7a: Unclear which signals denote nhr-114 and Y41C4A.32.

Version 1:

Reviewer comments:

Reviewer #2

(Remarks to the Author)
All my points were addressed.

Reviewer #3

(Remarks to the Author)
The authors have addressed almost all my concerns. I still have a minor comment.
1. Lines 194-195, "low Y41C4A.32 reporter levels of sams-1;rnp-6 worms can be attributed to the severe growth impairment of the double mutant (Figure 3i)).": The authors sate that the low reporter expression is attributed to the severe growth impairment. However, the evidence is not shown in the manuscript. The low expression "could" be attributed to the severe growth impairment considering the data and explanation in the rebuttal.

Open Access This Peer Review File is licensed under a Creative Commons Attribution 4.0 International License, which permits use, sharing, adaptation, distribution and reproduction in any medium or format, as long as you give appropriate

credit to the original author(s) and the source, provide a link to the Creative Commons license, and indicate if changes were made.
In cases where reviewers are anonymous, credit should be given to 'Anonymous Referee' and the source.
The images or other third party material in this Peer Review File are included in the article's Creative Commons license, unless indicated otherwise in a credit line to the material. If material is not included in the article's Creative Commons license and your intended use is not permitted by statutory regulation or exceeds the permitted use, you will need to obtain permission directly from the copyright holder.
To view a copy of this license, visit https://creativecommons.org/licenses/by/4.0/

Reviewer's Comments:

Reviewer #1 (Remarks to the Author)

Here, Huang et al. present a really thorough investigation of how RNP-6 regulates the expression of genes involved in S-adenosylmethionine and S-adenosylhomocysteine cycling and metabolism. In addition, the authors present the surprising findings that increased dietary B12 supplementation can alleviate many of the defects observed in their C. elegans rnp-6 mutant model. As part of these studies the authors conduct two independent and unbiased genetic screens, one in E. coli (C. elegans food source), and one in C. elegans itself. In both cases the authors identify genes that converge on vitamin B12 and methionine/SAM metabolism. These findings, coupled with substantial additional analyses and validation, make a convincing case that specific metabolic defects underlie many of the organismal level pathologies in rnp-6 mutant animals. The authors further build on these findings and demonstrate that they are not unique to rnp-6 mutant animals but that similar observations were observed for mutations in other splicing factors (prp-19) and thus were not specific to the rnp-6 mutant model. They also add some data that suggests, even if not conclusive, that human patient cell line models and humans with Verheij syndrome might have similar metabolic defects. Overall, I think this is a very well done study with substantial data. I think the data supports the claims made in the manuscript and its well written. As is clear in the model in Figure 7 some questions remain, however I think there is already substantial data in this manuscript as is and that the conclusions are strong and represent a significant advance in the field. Given the surprising and very interesting findings and the already rigorous approach. I do not feel that additional experiments are required for publication, which is not something I've been able to do often on a first review. Interesting work and very well done.
We thank the reviewer for their positive assessment. It is greatly appreciated!

Reviewer #2 (Remarks to the Author)

Comments on Figure 5
Wenming Huang et al Fig. 5 presents a coherent and logically structured set of experiments linking rnp-6–dependent splicing defects to functional loss of the transcription factor nhr-114. The progression from global splicing alterations to a specific causal intron retention event is well motivated, and the genetic rescue experiments provide strong support for the central conclusion that aberrant nhr-114 splicing is a major driver of rnp-6 mutant phenotypes. Overall, Figure 5 is a strong centerpiece of the manuscript and provides compelling genetic evidence linking spliceosomal dysfunction to metabolic pathology via nhr-114 mis-splicing.

Major points:
1. Scope of causality
As I understand nhr-114 promotes a change in splicing that is associated with the observed phenotype. The authors cherry pick one event to better connect the two. While the specific event chosen is well demonstrated as a target of nhr-114, some of the other targets can be non-direct. For this reason I would edit the text of "primarily caused" to something more subtle.
We thank the reviewer for highlighting causal nuance. The legend title now states that aberrant nhr-114 splicing "drives" rnp-6 defects, replacing "primarily caused" (Figure 5, Figure S6). This should emphasize our direct evidence while accommodating additional targets.

2. Fig. 1F shows a sashimi plot of the intron, one can also absorve that the intron upstream is also retained in response to nhr-114. This should be discussed.
Does crispr in panel k predicted to affect the retention of this intron?
We thank the reviewer for noting the intron 5 retention signal in Figure 5f. Indeed, this observation was confirmed by RT-PCR (see Figure 1 below). However, due to the moderate effect size, we did not purse this IR event futher. As suggested, we have highlighted this observation: "Additionally, read coverage spanned downstream intron 5, indicating concurrent retention (Figure 5f)." (Results, 370-371). We also clarified that intron 4 CRISPR edits target only that site, with no expected or assessed impact on intron 5: "These edits targeted solely the intron 4 3' splice site and thus were not expected to affect downstream intron 5." (Results, 401)

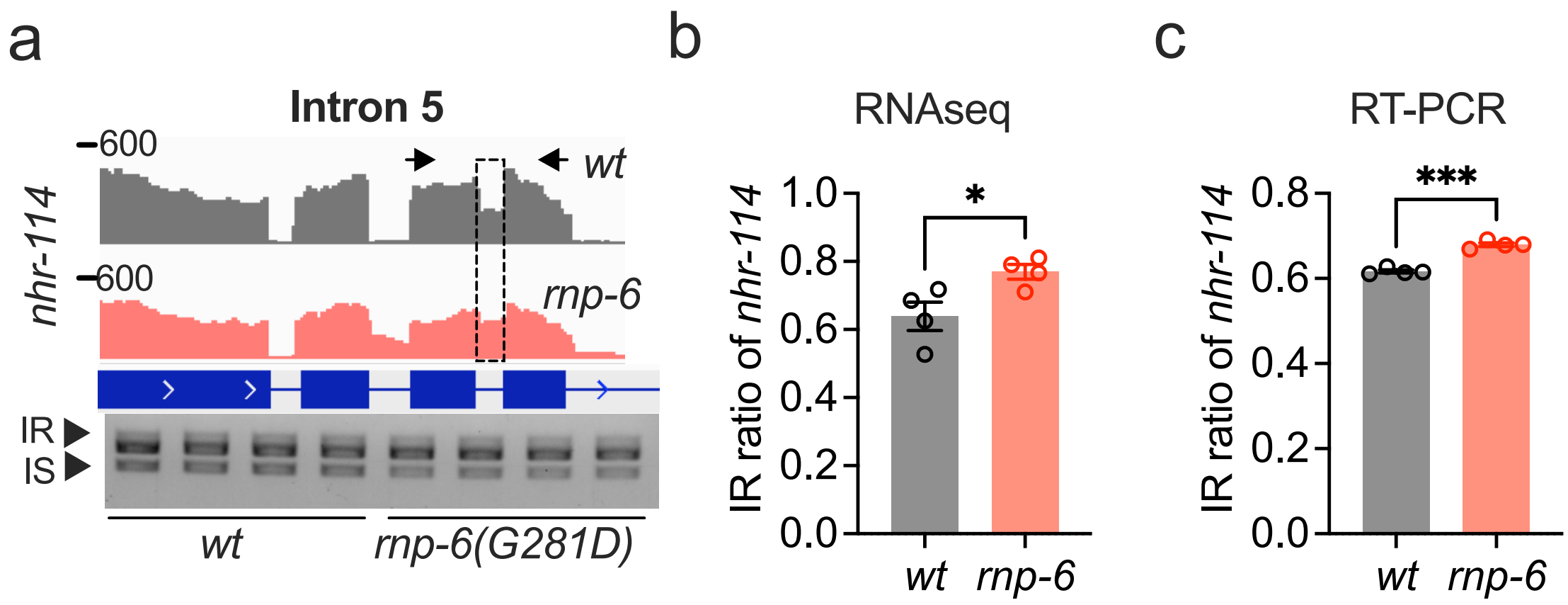

Figure 1. rnp-6(G281D) mutation causes moderate intron 5 retention in nhr-114 transcripts.

3. Protein-level validation
The reduction in GFP::NHR-114 signal (panels i–j) supports functional loss, but it is not entirely clear whether decreased fluorescence reflects reduced protein abundance, altered stability, or cell-type–specific effects. A brief clarification in the legend or text noting whether GFP tagging preserves normal localization and function would improve interpretability.
We thank the reviewer for suggesting clarification of the GFP::NHR-114 data. We have added a note to the legend, clarifying that the tag does not alter localization or function: "GFP tagging preserves wild-type localization and function." (Figure 5i–j)

Minor comments:
Many of the acronyms are not explained or explained many times: Verheij syndrome (OMIM #615583) VRJS, Vitamin B12, DSGs
We thank the reviewer for noting acronym usage and consistency. We have introduced Verhaij syndrome (VRJS), S-adenosylmethionine (SAM), S-adenosylhomocysteine (SAH), Vitamin B_{12} (VB12), phosphatidylcholine (PC), homocysteine (Hcy), one-carbon cycle (1C), tricarboxylic acid (TCA), intron retention (IR), differentially spliced gene (DSG), and differentially expressed gene (DEG) at first mention only and removed subsequent expansions in the main text. We further removed acronyms for small nuclear ribonucleoprotein particles (snRNPs), methionine synthase (MS), and mass spectrometry (MS) (removed in Results, but kept in Methods).

Reviewer #3 (Remarks to the Author)

In the manuscript, Huang et al. analyzed rnp-6(G281D) hypomorphic mutant of C. elegans and demonstrate that dysregulation of this splicing factor causes smaller body size and slower growth rate through imbalance in SAM and phosphatidylcholine metabolism.
First, they discovered that the phenotypes were restored by feeding with distinct E. coli strains and identified hundreds of differentially expressed and/or spliced genes on diet. They established a Y41C4A.32 reporter to read out the rnp-6(G281D)/OP50-specific condition and identified vitamin B12 (VB12) as sufficient to restore the phenotypes except for longevity. They also searched for host factors and identified methionine metabolism pathways, consistent with VB12 requirement. Metabolomic and lipodomic analyses revealed imbalances in SAM/SAH and phosphatidylcholine, and VB12 supplementation restored most of the lipid metabolism. They further screened direct RNP-6 targets and identified intron retention of nhr-114 as a primary cause of the rnp-6(G281D) phenotypes. They further demonstrate that mTORC signaling is affected in the mutant and is critical for phenotype restoration by VB12 supplementation. Mutations in the human ortholog of rnp-6, PUF60, is know to cause Verheij syndrome (VRJS) and the authors demonstrate that knockdown of PUF60 also affected splicing of a similar set of metabolism-related genes. Furthermore, metabolites and lipids are affected in VRJS patient bloods.
From these and other results the authors propose that spliceosomopathies, in both C. elegans and humans, can be alleviated with VB12.

Overall, the study is well organized, and the results are clearly described. The main conclusion of the study is well supported by the key experiments such as genome editing of the nhr-114 gene and use of the raga-1 (lof) mutant. I have a few concerns regarding the description in the main text.

Major criticisms:
1. Lines 195-196, "We found that deletion of sams-1 fully abolished the rescue by BW25113, VB12 and methionine in rnp-6(G281D) mutants (Figure 3h–i)": Figures 3h and 3j demonstrate that expression of the Y41C4A.32 reporter is suppressed by the sams-1 mutation in the rnp-6(G281D) mutant, and that feeding with BW25113 or supplementation with VB12 or methionine enhances its expression. If enough amount of SAM is critical for suppressing the reporter in the rnp-6(G281D) mutant, its expression would be higher in the sams-1 background fed with OP50. Please discuss this unexpected result.
We thank the reviewer for this comment. The deletion of sams-1 does not completely abolish SAM production (PMID: 22035958), as other sams genes (such as sams-3/4/5) also contribute to SAM synthesis (PMID: 36756948). We now say: "The incomplete effect of sams-1 likely reflects the residual activity of sams-3/4/5." (Results, 257)

Consistent with this, we observed that the loss of sams-1 significantly induces expression of the Y41C4A.32 reporter in the wild-type background (see Figure 2 below). However, sams-1 deletion exacerbates growth defects of rnp-6 mutants, causing markedly deleayed development and smaller body size in double mutants (manuscript Figure 3i), mimicking a synthetic growth delay phenotype. We reason that this severe growth impairment compromises Y41C4A.32 reporter expression. Supplementation with BW25113, VB12, or methionine modestly enhances SAM production and partially rescues worm growth (although not statistically significant), likely resulting in a modest increase of Y41C4A.32 reporter expression. We added to the main text: "Furthermore, low Y41C4A.32 reporter levels of sams-1;rnp-6 worms can be attributed to the severe growth impairment of the double mutant (Figure 3i)." (Results, 257–259)

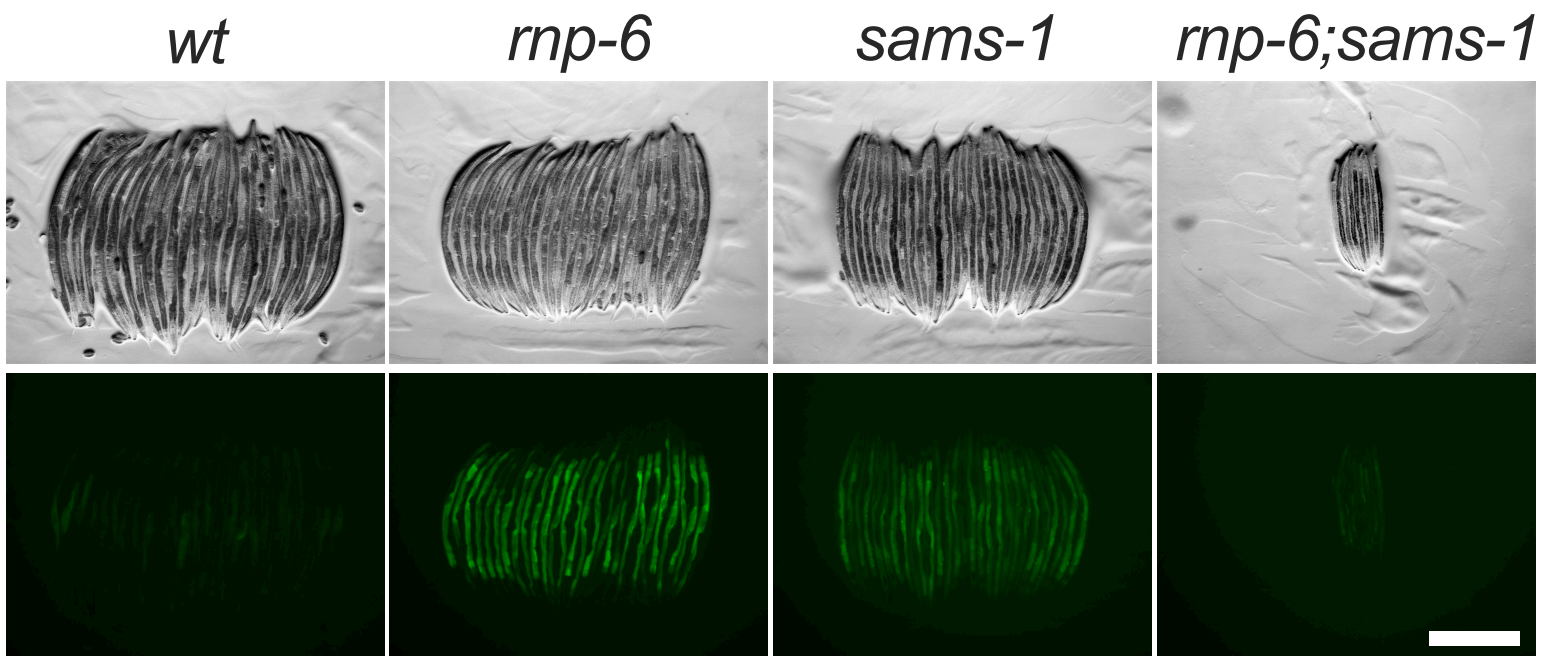

Figure 2. sams-1 deletion exacerbates growth defects and diminishes Y41C4A.32 reporter expression in rnp-6(G281D) mutants.

2. Lines 201-203, "These findings support a model in which SAM-dependent phosphatidylcholine synthesis constitutes a major downstream mechanism by which K12-type E. coli and VB12 rescue rnp-6 mutant phenotypes.": Even though choline supplementation bypassed the requirement for sams-1 in the rnp-6(G281D) mutants (Figure 3j–k), the results does not necessarily indicate that phosphatidylcholine is the critical downstream mechanism for rescue as in the title; choline supplementation may have just attenuated consumption of SAM.

We appreciate the critical point raised by the reviewer. We have modified the phrasing to better reflect our findings: " These findings are consistent with a model in which SAM-dependent PC synthesis contributes to the rescue of rnp-6 mutant phenotypes by K12-type E. coli and VB12, though we cannot exclude the possibility that choline supplementation also promotes rescue by altering SAM availability." (Results, 263–266)

3. Lines 305-306, "~60% (288/483) of the shared genes were rescued by VB12 supplementation in the nhr-114 mutant and by BW25113 feeding in the rnp-6 mutant (Figure S6h, Table S10)": Please check if this description is correct. I understand there are 573 genes rescued by BW25113 feeding in the rnp-6 mutant (Figure S1h). The source of the RNA-seq data for the nhr-114 mutant supplemented with VB12 is not mentioned.

We thank the reviewer for pointing this out. The 483 genes were the overlapped genes dysregulated by both rnp-6 and nhr-114 mutations on OP50. Of these, 288 genes were rescued by both VB12 supplementation in nhr-114 mutants and BW25113 feeding in rnp-6 mutants. These 288 genes constitute a subgroup of the 573 genes rescued by BW25113 in rnp-6 mutants. The VB12-supplemented nhr-114 RNA-seq dataset derives from the same resource as the unsupplemented nhr-114 data. We have clarified this information in both main text and "Data availability" section. (Results, 379; Data availability)

Minor points:

1. Lines 104-105, "Feeding with BW25113 significantly changed the expression of 1319 genes in rnp-6 mutants": It seems from Figure S1h that there are 1419 DEGs.

The correct number is 1419. We have updated the main text accordingly. (Results, 145)

2. Lines 105-107, "WormCat 2.0 gene set analysis of the BW25113-restored genes revealed significant enrichment in ... (Figure 1f).": The main text mentions analysis on 573 of "BW25113-restored" genes whereas Figure 1f analyzes 1319 of "BW25113-responsive" genes. Please clarify which is correct.

We thank the reviewer for spotting this inconsistency. We have corrected the figure legend accordingly. (Figure 1f)

3. Line 264, "most SE events (217 out of 227 events) decreased": It is confusing whether exon skipping was decreased or exon inclusion was decreased.

We have changed the text to clarify the changes: “Among 638 significant splicing alterations, intron retention (IR) and cassette exon (SE) were the most prevalent (Figure 5a). Nearly all IR events (315 out of 330 events) showed increased intron retention, whereas most SE events (217 out of 227 events) exhibited reduced exon inclusion (Figure 5b), confirming a widespread disruption of canonical splicing in rnp-6(G281D) mutants.” (Results, 341–349)

4. Line 284, “nhr-114 showed the strongest effect on both phenotypes (Figure 5d–e, S6b–d)”: Figure S6d may not be related to this claim.
We have corrected the figure reference in the main text to “S6b–c”. (Results, 367)

5. Lines 297-298, “483 of these genes were also significantly altered in rnp-6(G281D) (overlap significance, $p < 5e-12$) (Figure S6e–f; Table S10).”: Figure S6f may not be related to this claim.
We thank the reviewer for pointing this out. We have corrected the figure reference in the main text to “S6e”. (Results, 383)

6. Lines 303-304, “Less than 4% (18/483) of the overlapped differentially expressed genes displayed aberrant splicing in rnp-6 mutants (overlap significance, $p < 0.102$) (Table S10)”: Figure S6f may also support this claim.
We have changed the text from “(Table S10)” to “(Figure S6f, Table S10)” (Results, 388)

7. Line 313, “nhr-114(i4+) phenocopied rnp-6(G281D)”: Please clarify if this i4+ knock-in allele is homozygous or heterozygous.
Both nhr-114(i4+) and nhr-114(i4-) mutants are homozygous. We have clarified this in the main text. (Results, 403 and 406)

8. Lines 314-315, “while not further elevating Y41C4A.32 expression in rnp-6(G281D) mutants (Figure 5m–o)”: It seems from Figure 5l-m that Y41C4A.32 expression is elevated in rnp-6(G281D) mutants. I think this makes sense if the i4+ allele is homozygous. Figure 5l is not cited in the main text.
We thank the reviewer for spotting this error. The si4+ allele further enhance the expression of Y41C4A.32 reporter expression in rnp-6 mutants. We have corrected the text accordingly and cited Figure 5l. (Results, 405; Figure 5l)

9. Lines 346-347, “Under ARD conditions nhr-114 intron 4 retention was significantly increased by ~10% relative to fed controls (Figure S7k) accompanied by reduced levels of nhr-114 mRNA (Figure S7l).”: These claims should cite Figure S7j and S7k, respectively. Figures S7j-k compare ARD conditions with Recovery conditions and lack normal conditions.
We thank the reviewer for pointing this out. We have corrected the citation. S7k and S7l should be S7j and S7k, respectively. (Results 436–437)

10. Lines 393-394, “Although the levels of PC remained unchanged, the levels of PE and the ratio of PC/PE were slightly increased (Figure 7d).”: Figure 7d demonstrates that the level of PE was slightly decreased.
We have corrected the main text by changing it to “Although the levels of PC remained unchanged, the levels of PE decreased and the ratio of PC/PE was slightly increased (Figure 7d).” (Results, 495)

11. Figure 1b-c, g and many others: Please make it clear in the legend that each open circle represent the average of multiple worms in a set of experiment. “Data are presented as

mean ±s.e.m.": Please clarify whether the mean and the s.e.m. are those of all worms or of the averages.
We thank the reviewer for suggesting this important clarification. We have revised all relevant figure legends to state: "Each open circle in a bar graph represents the average value of multiple worms in one experimental replicate. Data are presented as mean ± s.e.m. of averages unless otherwise indicated. "n" denotes experimental replicates, with animal counts summarized in each bar graph." (Figure legends)

12. Legend to Figure 1g, "F.C., fold change": Not involved in the Figure.
We have updated the y-axis label of Figure 1g to "Relative mRNA level of Y41C4A.32 (F.C.)" and revised the legend accordingly. (Figure 1g)

13. Figure 2d: Not cited in the main text.
We thank the reviewer for identifying this oversight. We have added the citation to the main text: "Subsequent phenotypic analysis revealed that deletion of btuF or tonB in BW25113 also mimics OP50 E. coli effects, resulting in smaller body size of rnp-6 mutants (Figure2d, Figure S2a-b)" (Results, 190)

14. Figure 3f-g and j-k: Please clarify that these worms were fed with OP50.
We have revised the information in figure legend as follows: "f–g, Effect of methionine supplementation (10 mM) on reporter expression (f) and body size (g) of rnp-6;metr-1 double mutants grown on OP50 bacteria (n = 4)." (Figure 3f–g, j–k)

15. Legend to Figure 6c, "Scale bar, 20 mm.": This may be micrometer.
Thanks for pointing this out. We have corrected it. (Figure 6c)

16. Figure S1, lines 4-5, "bars represent the ratio of alive to dead worms": Methods section describes "Cold survival ratio was measured as the ratio of the number of live worms to the number of total worms." Please make sure which is correct.
The bars represent the ratio of alive to total worms. We have corrected this in the figure legend. (Figure S1a)

17. Figure S1, line 5, "diet after cold shock": It seems from the Method section that the E. coli diet was NOT used after cold shock.
We thank the reviewer for pointing out this ambiguity. For the cold tolerance assay, the cold shocked worms were recovered on plates seeded with E. coli (OP50 or BW25113) supplemented with or without compounds. We have clarified this detail in the Methods section. (Methods, Cold tolerance assay)

18. Figure S1m: Unclear what the y-axis indicates.
We thank the reviewer for pointing this out. The y-axis in Figure S1m indicates the ratio of bigger isoform to smaller isoform for tcer-1 and tos-1, as shown in Figure S1l. We have added this information to the figure legend. (Figure S1m)

19. Figure S2j-k: Please explain what "YD" indicates.
"YD" denotes young adult stage. We have added the information to the figure legend. (Figure S2j–k)

20. Figure S7a: Unclear which signals denote nhr-114 and Y41C4A.32.
We have added arrowheads to indicate the signals for nhr-114 and Y41C4A.32. (Figure S7a)

Reviewers' Comments

Reviewer #2 (Remarks to the Author):

All my points were addressed.

Reviewer #3 (Remarks to the Author):

The authors have addressed almost all my concerns. I still have a minor comment.
1. Lines 194-195, "low Y41C4A.32 reporter levels of sams-1;rnp-6 worms can be attributed to the severe growth impairment of the double mutant (Figure 3i)).": The authors sate that the low reporter expression is attributed to the severe growth impairment. However, the evidence is not shown in the manuscript. The low expression "could" be attributed to the severe growth impairment considering the data and explanation in the rebuttal.

We thank the reviewer for pointing this out. We updated the main text accordingly: "The incomplete effect of sams-1 loss likely reflects residual activity of sams-3/4/5. Furthermore, low Y41C4A.32 reporter levels of sams-1;rnp-6 worms could be attributed to the severe growth impairment of the double mutant (data not shown)." (Results, 195-197)
