# Supplementary material for: Vitamin B12 alleviates spliceosomopathy via phospholipid remodeling
Source: Nat Commun. 2026 Aug 7;17:8011. doi: 10.1038/s41467-026-76295-9 (PMC13451374; doi:10.1038/s41467-026-76295-9)
Supplement: Supplementary file 4 [file 41467_2026_76295_MOESM4_ESM.pdf]

Description of Additional Supplementary Files

File Name: Supplementary Data 1

Description: Gene expression analysis of rnp-6(G281D) mutants.

File Name: Supplementary Data 2

Description: Gene splicing analysis of rnp-6(G281D) mutants.

File Name: Supplementary Data 3

Description: List of overlapping differentially expressed genes (DEGs) and differentially spliced genes (DSGs) of rnp-6_OP50 vs wt_OP50.

File Name: Supplementary Data 4

Description: List of DEGs (rnp-6_OP50 vs wt_OP50) that are significantly rescued by E. coli BW25113 diet.

File Name: Supplementary Data 5

Description: List of E. coli BW25113 mutants that (partially) lose the rescue effects on cpbl-1/Y41C4A.32 reporter expression.

File Name: Supplementary Data 6

Description: Metabolomic analysis of rnp-6(G281D) mutants.

File Name: Supplementary Data 7

Description: Lipidomic analysis of rnp-6(G281D) mutants.

File Name: Supplementary Data 8

Description: List of metabolism-related genes that show robust splicing changes in rnp-6(G281D) mutants.

File Name: Supplementary Data 9

Description: Hits from targeted RNAi-knockdown screen of DSGs (rnp-6_OP50 vs wt_OP50).

File Name: Supplementary Data 10

Description: Gene expression analysis of nhr-114 mutants.

File Name: Supplementary Data 11

Description: Gene expression analysis of prp-19 RNAi.

File Name: Supplementary Data 12

Description: Gene splicing analysis of prp-19 RNAi.

File Name: Supplementary Data 13

Description: Gene splicing analysis of PUF60 siRNA-treated PC9 cells.

File Name: Supplementary Data 14

Description: KEGG pathway analysis of PUF60-dependent DSGs.

File Name: Supplementary Data 15

Description: Metabolomic analysis of Verheij Syndrome patient plasma.

File Name: Supplementary Data 16

Description: Lipidomic analysis of Verheij Syndrome patient plasma.

File Name: Supplementary Data 17

Description: Reagents and resources used in this study.

File Name: Supplementary Data 18

Description: Lifespan results generated in this study.
